# Encapsulation of *Berberis vulgaris* Anthocyanins into Nanoliposome Composed of Rapeseed Lecithin: A Comprehensive Study on Physicochemical Characteristics and Biocompatibility

**DOI:** 10.3390/foods10030492

**Published:** 2021-02-25

**Authors:** Mina Homayoonfal, Seyed Mohammad Mousavi, Hossein Kiani, Gholamreza Askari, Stephane Desobry, Elmira Arab-Tehrany

**Affiliations:** 1 Bioprocessing and Biodetection Lab (BBL), Department of Food Science and Technology, University of Tehran, Karaj 999067, Iran; minahomayoonfal@ut.ac.ir (M.H.); mousavi@ut.ac.ir (S.M.M.); hokiani@ut.ac.ir (H.K.); iraskari@ut.ac.ir (G.A.); 2 Laboratoire D’Ingénierie des Biomolécules (LIBio), Université de Lorraine, 2 Avenue de la Forêt de Haye—TSA 40602, CEDEX, 54518 Vandoeuvre-lès-Nancy, France

**Keywords:** barberry, anthocyanin, nanoliposome, encapsulation efficiency, biocompatibility

## Abstract

In the present study, nanoliposomes composed of rapeseed lecithin were used for the encapsulation of anthocyanin compounds (AC). The nanoliposomes were prepared using hydration and ultrasound combined method, and the effect of AC concentration (4.5, 6.75, 9% *w*/*w*) on the characteristics of nanoliposomes including particle size, polydispersity index (PDI), zeta potential, and the encapsulation efficiency (EE) of nanoliposomes with and without AC were studied. The results suggested the fabricated nanoliposomes had a size range of 141–196 nm, negative zeta potential and narrow particle size distribution. Further, the samples containing 9% extract had the maximum EE (43%). The results showed elevation of AC concentration resulted in increased particle size, PDI, EE, and surface charge of nanoparticles. The presence of AC extract led to diminished membrane fluidity through the hydrophobic interactions with the hydrocarbon chain of fatty acids. TEM images suggested that the nanoliposomes were nearly spherical and the AC caused their improved sphericity. Further, in vitro biocompatibility tests for human mesenchymal (MSC) and fibroblast (FBL) cells indicated nanoparticles were not toxic. Specifically, the best formulations with the maximum compatibility and bioavailability for MSC and FBL cells were AC-loaded nanoliposomes with concentrations of 0.5 mL/mg and 10.3 mL/µg and, respectively.

## 1. Introduction

Barberry (*Berberis vulgaris* UKSI) is an evergreen plant belonging to Berberidaceae family. It is cultivated in Europe, Asia and especially Iran [1]. Barberry is one of the herbal plants whose extract enjoys different biological characteristics including anti-inflammatory, anti-hypertensive, and anti-arrhythmia effects [2]. These features are related to its constituent phytochemical components including antioxidant, phenolic, and anthocyanin compounds [3]. Anthocyanin compounds (AC) are the dominant species of phenolic compounds in barberry and naturally play a significant role in developing its pharmacological and health-promoting effects [4]. These antioxidants are able to prevent the incidence of cardiovascular and neurological disease as well as cancer and diabetes [5]. However, these compounds are sensitive to environmental conditions and more importantly, phenolic hydroxyl groups of AC are easily oxidized to quinone, causing a reduction in their biological activity [6]. Hence, low stability and bioavailability have limited their usages in different processes. 

In recent years, different pharmaceutical and food industries have been facing serious challenges about preserving the quality of these bioactive compounds and enhancing their bioavailability. Accordingly, various strategies have been proposed, with encapsulation being the most applicable technique. Fundamentally, this method causes protection and elevation of stability, bioavailability, as well as targeting, controlled or long-term release of bioactive compounds through attaching them inside a coating or a thin layer wall as micro or nanoparticles.

Nanoliposomes are among the versatile methods of encapsulations in pharmaceutical, biological, and biochemical processes thanks to their variety of structure and composition [7]. Phospholipids, as the most important constituent of liposomes, are amphiphilic molecules composed of polar heads (hydrophilic or water-soluble parts) along with hydrophobic or lipid-soluble tail parts [8]. Liposomes are closed and spherical structures composed of bilayer lipids that are formed spontaneously in aqueous environments and encapsulate part of the surrounding solvent inside them. This characteristic causes the development of unique characteristics of liposomes, self-sealing, and it has attracted a great deal of attention in different industries including tissue engineering, pharmaceuticals, cosmetics, and food industries as favorable carrier systems [9,10]. Liposomes allow for encapsulating water and lipid-soluble compounds through entrapping them inside the internal core of liposomes and placement in the hydrophobic part, respectively. These transfer systems cause increased bioavailability and incidence of therapeutic activities owing to protecting bioactive compounds against the acidic environment of the stomach and increasing their absorption in the gastrointestinal tract [9,11]. 

So far, only a few studies have used liposome vectors for coating AC. Gibis et al., utilized soybean lecithin alongside high-pressure homogenization, while Zhao et al. used soybean lecithin coupled with supercritical fluid for encapsulation of phenolic compounds [12,13]. Similarly, Bryla et al. used soybean, sunflower, and egg lecithin along with a thin lipid film hydration method to the encapsulation of elderberry extract [8]. Rapeseed lecithin has three major types of mono and polyunsaturated fatty acids including oleic, linoleic, and linolenic acids. The last two components are essential fatty acids which cannot be produced by the body and play an important role in human health [10]. 

The aim of the present study was to develop a novel method for encapsulation of a barberry extract rich in AC within the structure of liposomes using rapeseed phospholipids and to evaluate the physicochemical characteristics of the nanoliposomes containing AC. In addition, the biological activity of AC extract and nanoliposomes was evaluated before and after loading of bioactive compounds in in vitro tests using human mesenchymal and fibroblasts stem cells. In this regard, the effect of concentration of AC extract and nanoliposomes (loaded and unloaded) was also studied on the cytotoxicity, metabolic activity and proliferation of the mentioned cells. 

## 2. Materials and Methods

### 2.1. Materials

Fresh barberry fruits were purchased from the Birjand region, Iran. In order to keep the quality, they were kept at room temperature and shade dried. Rapeseed lecithin was produced in the laboratory using a low-temperature enzymatic process without using any organic solvent [14]. Acetic acid, acetonitrile, BF_3_/methanol, chloroform, methanol, hexane, 1-(4-Trimethylammonium-phenyl)-6-phenyl-1,3,5-hexatrien (TMA-DPH), Dimethyl sulfoxide (DMSO), [4,5 dimethylthiazol-2-yl] 2,5 diphenyltetrazolium bromide (MTT) were supplied from Sigma-Aldrich (France) and Fisher Scientific (France). Hoechst 33528 and calf thymus DNA solutions were purchased from Invitrogen, molecular probes (France). Lactate dehydrogenase (LDH) cytotoxicity detection kit was obtained from Roche, Cat No. 11644793001, Sigma-Aldrich (France). All of the chemicals were analytical reagents.

Also, cell culture media and reagents included: alpha Minimal Eagle Medium (α-MEM, Lonza, Switzerland), Dulbecco’s Modified Eagle Medium (DMEM) low glucose (Gibco, France), Foetal Calf Serum (FSC, Sigma-Aldrich, France), L-glutamine (Gibco, France), Penicillin/Streptomycin (Gibco, France) and Fungizone ® (Gibco, France).

### 2.2. Preparation of AC Extract of Barberry

To prepare the AC extract, once cleaned and washed, barberry fruits were mixed with ultrapure water with 1:5 ratio respectively (i.e., 1 g barberry was mixed by 5 g water). Then, they were stirred for 5 min using Ultra-Turrax (T-25 Basic, IKA-Werk, Staufen, Germany) at 12,000 rpm. In order to remove solid impurities and fruit particles, the prepared mixture was size fractioned using vacuum filtration with cellulose filters of pore sizes 10, 8, 3, 1, 0.45 and 200 µm. Eventually, the filtered extract was concentrated in darkness using a vacuum rotary evaporator up to Brix 45 (i.e., grams of dissolved solid content/grams of solution was 45).

### 2.3. Production and Chemical Analysis of Rapeseed Lecithin 

Preparation of fatty acid methyl eaters (FAMEs) was conducted based on methods of Ackman et al., (1998) [15]. To identify fatty acids, gas chromatography equipped with flame ionization detector was utilized. Capillary column made of melted silica (length: 60 m, internal diameter: 25.0 mm, and thickness: 2.0 µm) was used to separate fatty acid methyl esters. The temperature of 250 °C was set for both the injector and detector. The temperature program of the column was adjusted as follows: initial temperature: 120 °C for 3 min, temperature rise to 180 °C at the rate of 2 min/°C and maintenance at this temperature for 10 min, temperature elevation (with the previous rate) to 220 °C for 25 min. To identify fatty acids, standards containing a combination of fatty acids were used (Polyunsaturated fatty acids (PUFA) from vegetable sources; Supelco, Sigma-Aldrich, Bellefonte, PA, USA). The obtained results were the average of three replications ± SD.

The lipid class of rapeseed lecithin was determined using thin-layer chromatography technique and Iatroscan MK-5 TLC-FID device (Iatron Laboratories Inc., Tokyo, Japan) equipped with a flame ionization detector. The separation and spotting of lipids were performed in Chromarod S-III silica. The samples were spotted automatically on the beginning point of Chromarod, and then the rods were placed inside a solvent tank. The rods were exposed to progressive phase solvent with the composition of hexane/diethyl ether/formic acid (80:20:2.0 *v*/*v*/*v*), then dried in an oven at 100 °C for 1 min, and eventually scanned by Iatroscan analyzer. The performance of Iatroscan occurred under the flow rates of 160 min/mL and 2 min/mL for hydrogen and air, respectively. In the next stage, to identify the polar lipids, the second progressive polar phase containing a mixture of chloroform, methanol, and ammonia was used with the following ratio (65:35:5 *v*/*v*/*v*). Finally, the obtained peaks were recorded and interpreted by the internal software ChromStar [10]. The results were expressed as the mean of 10 replications ± SD.

### 2.4. Preparation of Nanoliposomes

**Figure 1:**
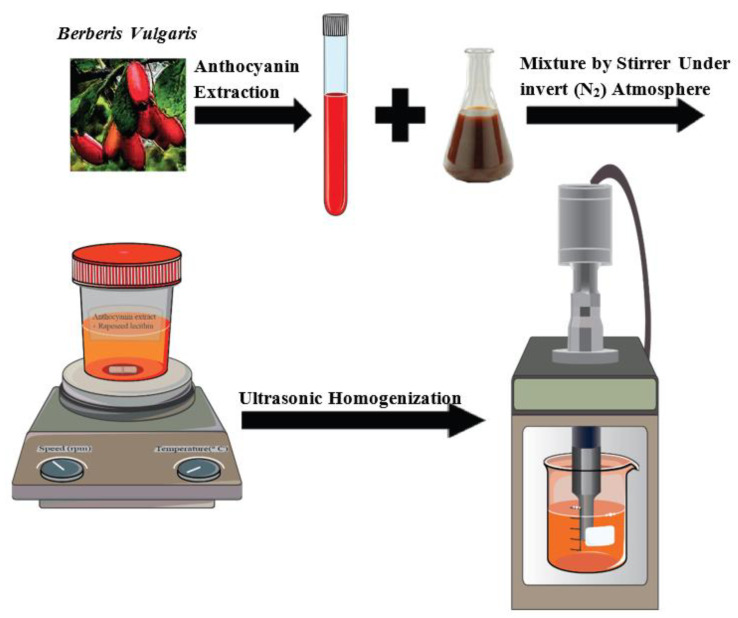
Schematic illustration of preparation of anthocyanin compounds (AC) incorporated in rapeseed nanoliposome.

In order to prepare the nanoliposome samples, concentrated barberry AC extract was mixed with deionized water at different ratios (4.5, 6.75, and 9% *w*/*v*). After adjusting the pH, rapeseed lecithin was added to the solution by 3 *w*/*v*%. Then, the prepared mixture was stirred for 5 h under inert atmospheric conditions (nitrogen). In the next stage, in order to obtain the colloidal suspension of nanoliposomes, the samples were sonicated using an ultrasound homogenizer (probe with a diameter of 13 mm for samples with the volume of 50 mL) at the frequency of 40 kHz and power of 40% for 4 min (1 s on, 1 s off). The unloaded nanoliposomes were prepared exactly using the mentioned stages but without AC extract [9]. Schematic illustration of ACs incorporated in rapeseed nanoliposome is represented in Figure 1.

### 2.5. Characterization of Nanoliposomes with and without Active Molecule

#### 2.5.1. Determining the Particle Size of Nanoliposomes

The mean of hydrodynamic diameter and particle size distribution of vesicles were determined using the dynamic light scattering (DLS) technique (Malvern Zetasizer Nano ZS, Malvern Instruments, UK). In order to enhance the distance between particles, prevent the multiple scattering effects and false results, the sample was diluted with ultrapure water with a ratio of 1:400 and placed inside a disposable folded capillary cuvette. The intensity of scattering was calculated at the scattering angle of 173° and at 25 °C by an avalanche photodiodes detector. In order to obtain the distribution of the transmission diffusion coefficient of the particles (an important parameter which is associated with the hydrodynamic diameter of particles through Stoke–Einstein relation), the autocorrelation intensity function was analyzed by general-purpose algorithm added on in Zetasizer Malvern software. In the end, the particle size was calculated based on the data obtained by correlation function using dispersion technology software (DTS) and different algorithms [7]. In all tests, the refraction index (RI) and absorbance of samples were adjusted at 1.471 (as the standard value used for measuring the particle size of liposomes [10]) and 0.01, respectively. Further, all of the measurements were performed with three replications, and the results were reported as mean ± SD.

#### 2.5.2. Determination of the Zeta Potential of Nanoliposomes

Zeta potential of the samples was measured by Doppler laser electrophoresis technique and Malvern Zetasizer Nano ZS (Malvern Instruments, UK) to evaluate the net surface charge around the particles. In this regard and to prevent the multiple scattering effect, that samples were filtered, diluted by ultrapure water (1:400) and placed inside capillary electrophoresis cuvette with copper electrode [9]. Analysis of the samples was performed at 25 °C (4 replications for each sample), and the results were reported as mean ± SD.

#### 2.5.3. Encapsulation Efficiency (EE)

To calculate the encapsulation efficacy (EE) of AC in the prepared nanoliposomes, two identical samples of each AC-loaded nanoliposome (i.e., samples with 4.5, 6.75, and 9 *w*/*v*% AC) were considered: one sample for measuring the unencapsulated AC concentration and the other one for measuring the total AC concentration in the anthocyanin-loaded nanoliposomes. In this way, for estimating unloaded AC, the prepared nanoliposome samples were centrifuged for 45 min at 50,000 rpm and 4 °C (Optima ultracentrifuge, Beckman Coulter, Fullerton, CA, USA). At this stage, two separate phases were formed, the unencapsulated AC accumulated in the top phase, while the nanoliposomes aggregated in the bottom phase. In order to remove the trivial lecithin compounds which may not have been separated by applying centrifugal forces, the top phase was separated and mixed with chloroform at the ratio of 1:1, and once stirred, they were centrifuged at 10,000 rpm and 4 °C for 10 min. After the second centrifuge step, the top aqueous phase, included only water and AC, once was diluted with potassium chloride buffer (0.025 M, pH 1.0) and once sodium acetate buffer (0.4 M, pH 4.5) and finally, the concentration of AC was measured according to Equation 1 and regarded as unencapsulated ACs. For calculating the total AC concentration, the other sample was analyzed. In this step, AC-loaded nanoliposomes were broken down through mixing with chloroform at the ratio of 1:1, and the other steps were conducted similar to the first sample (after mixing with chloroform) and the total AC concentration in the sample was calculated according to Equation (1).

(1) $$\mathrm{AC}\;\mathrm{concentration}\;\left(\mathrm{mg}/L\right)=\;\frac{\left({\left(A_{510}-A_{700}\right)}_{\mathrm{pH}\;1}-{\left(A_{510}-A_{700}\right)}_{\mathrm{pH}\;4.5}\right)\times \mathrm{MW}\times \mathrm{DF}\times 1000}{\varepsilon \times L}$$

In this equation, A and its index represent the absorbance and wavelength respectively, MW is molecular with of cyanidin 3-glucoside (449.2 g/mole), DF indicates dilution factor, ε is molar absorbance of cyaniding 3-glycoside (26,900) and L indicates cell path length (1 cm). All absorbance measurements were conducted by a UV-Visible spectrophotometer (HACH-DR/4000U, USA)

(2) $$\mathrm{Encapsulation}\;\mathrm{efficacy}\;\left(\%\right)=\;\frac{\mathrm{Total}\;\mathrm{AC}\;\mathrm{concentration}-\mathrm{unencapsulated}\;\mathrm{AC}\;\mathrm{concentration}}{\mathrm{Total}\;\mathrm{AC}\;\mathrm{concentration}}\times 100$$

#### 2.5.4. Stability of Nanoliposomes

The stability of the nanoliposome samples with and without AC was examined at 4 °C and 37 °C for 30 days. The samples were placed inside a dry chamber, and the particle size, polydispersity index (PDI), zeta potential, and EE were evaluated every ten days.

#### 2.5.5. Transmission Electron Microscopy (TEM)

To investigate the morphology and microstructure of nanoliposomes, transmission electron microscope (TEM) and negative staining methods were used. For this purpose and to decrease the effect of concentration, the samples were diluted 30 times with distilled water. In the next stage, the same volume of the diluted sample and 2% ammonium molybdate solution were mixed with each other and subject to environmental conditions for 3 min. A drop of the sample was placed on a copper grid (mesh 200, diameter 300 mm) protected by formvar-carbon for 5 min. The extra amounts of the sample were collected by filter paper and to dry the samples, the grids were exposed to room temperature for 5 min. The micrographs were developed using transmission electron microscope (CM20 Phillips) operating at 200 kV, and then recorded by Olympus CCD TEM Camera [16].

#### 2.5.6. Membrane Fluidity

The membrane fluidity of nanoliposomes was determined by fluorescence polarization and determining TMA-DPH fluorescent level. TMA-DPH is a compound containing cationic group of trimethyl ammonium (TMA), which functions as a superficial barrier to improve the establishment of fluorescence probe (DPH) inside the membrane. Accordingly, first, TMA-DPH solution was prepared in ethanol with a concentration of 1 mM and added to the prepared nanoliposomes, and a final concentration of 4 µM and 2.0 mg/mL was obtained for the probe and lipid, respectively. The resultant mixture was stirred gently at room temperature and in darkness for 1 h, and then poured into black microplates (180 µL inside each well). TMA-DPH and nanoliposome samples interacted with each other, and the fluorescence probes were aligned horizontally and vertically inside the lipid bilayers. The fluorescence intensity of each sample was measured by Tecan INFINITE 200R PRO (Austria) equipped with fluorescence polarizers. The samples were excited at 360 nm and emitted at 430 nm under constant stirring at 25 °C. The data were analyzed by Magellan 7 software (Tecan Group Ltd., Switzerland). The extent of polarization of TMA-DPH (p) was considered by the following equation:

(3) $$P=\frac{I_{∥}-{\mathrm{GL}}_{⊥}}{I_{∥}+2{\mathrm{GI}}_{⊥}}$$

In this relation, I_║_ and I_┴_ represent the horizontal and perpendicular fluorescent intensity to the excitation plane respectively, and G is a factor related to the transmission efficiency. The membrane fluidity was defined as inverse of polarization (1/P) [17].

### 2.6. In Vitro Biocompatibility Tests

The in vitro tests were conducted by mesenchymal (MSC) and fibroblast (FBL) stem human cells. Preparation and cultivation of MSC and FBL cells were performed in DMEM culture medium. Furthermore, the AC extract (Ext) and unloaded nanoliposomes (FL) and AC-loaded nanoliposomes (Encap) were added at different concentrations (1 mg.mL^-1^, 0.5 mg.mL^−1^, 20.7µg.mL^−1^, and 10.4 µg.mL^−1^) to the cell culture medium. In order to investigate the effect of the aforesaid treatments on cellular function, cellular cytotoxicity (LDH analysis), metabolic activity (MTT analysis) and cell proliferation (DNA content, Hoechst analysis) were evaluated. Eventually, for LDH and MTT tests, the cells were cultured in plates containing 96 wells, and for the proliferation test, they were cultured in plates containing 12 wells. The number of cells for each test was 1000 cell/cm^2^. LDH, MTT and DNA content tests were performed at specific time point: D1 (Day 1), D3 (Day 3) and D7 (Day 7). Further, a group of cells that had been grown only in the presence of the culture medium was considered as the control sample (CTL). 

#### 2.6.1. Cellular Cytotoxicity Evaluation by LDH Assay

Lactate dehydrogenase (LDH) is an intracellular enzyme existing in cytoplasm of healthy cells. If this enzyme is released to a large extent from the cell, it signals cytotoxicity of the environment and the substance. For this reason, integration of the cellular membrane was determined by measuring LDH level secreted to outside the cell according to the manufacturer’s instructions (LDH dehydrogenase activity assay kit, Sigma Aldrich, Munich, Germany). For this purpose, the cells were centrifuged for 10 min at 300 g, then 100 µL of the top phase liquid was transferred to the 96-well plates, to which 100 µL of LDH reagent was added. The resulting mixture was gradually stirred away from light for 30 min, and in the end, the absorbance was measured at 490 nm by spectrophotometer (Varioskan ® Flash, Thermo Fisher Scientific Inc., Essone, France) to determine the reduced content of nicotinamide adenine dinucleotide (NAD) in presence of LDH [18].

#### 2.6.2. Cellular Metabolic Activity Assessment by MTT Test

MTT test was performed to evaluate the metabolic activity of cells. The cells in their active state can convert MTT to formazan. MTT as a yellow-colored salt of tetrazolium is reduced to purple formazan in response to the functioning of reductase succinate-tetrazolium system belonging to the mitochondrial respiratory chain with concurrent oxidation of NADH and NADPH. For this purpose, 50 µL of MTT solution was added to 200 µL of the cellular culture medium. Then was incubated for 4 h at 37 °C and atmosphere containing 5% CO_2_, and 95% humidity. Under these conditions, the yellow color of MTT solution was converted to blue crystals of formazan as the result of cellular metabolism and mitochondrial dehydrogenase. Next, the top phase was removed and the solid crystals were converted to a soluble state by 200 µL of DMSO reagent, which was then gradually stirred at 37 °C for 5 min. The top phase was separated and the concentration of formazan was measured by spectrophotometer (absorbance at 540 nm) [19].

#### 2.6.3. Cell Proliferation Estimation by Hoescht Assay 

In order to evaluate the cellular proliferation, DNA content was measured. The basis of this experiment involves the ability to attach Hoescht 33258 as the fluorescent color to DNA. Thus, to sample preparation, the cells were washed three times with phosphate-buffered saline (PBS), trypsinized with a solution of trypsin-EDTA (0.25%), and centrifuged at 300 g for 10 min. Next, the samples were mixed with 200 µL of Hoechst 33258 buffer. In order to DNA release, cells were degraded by three consecutive cycles of freezing (by liquid nitrogen) and defrosting (in a water bath (37 °C)). Then, the volume of each sample was brought to 1 mL by Hoechst 33,258 solution, and their absorption was measured by fluorescence spectrophotometer (360 nm excitation and 430 nm emission) [20].

### 2.7. Statistical Analysis

In this study, all experiments were conducted in triplicate and all experimental data in this study (in the tables and the context) were presented as mean ± standard deviation (SD). Statistical analyses of data were performed by Statistical Product and Service Solutions (SPSS) 23.00 (IBM, Armonk, NY, USA). Accordingly, analysis of variance (ANOVA) was accomplished for all results and *p* < 0.05 was regarded as significant.

## 3. Results and Discussion

### 3.1. Rapeseed Lecithin Fatty Acid Composition and Lipidic Class

The profile of the main fatty acid composition constituting rapeseed phospholipids is presented in Table 1. As observed, monounsaturated fatty acids (MUFA) with a total content of 58.89% are the major lipid compounds of rapeseed lecithin. Meanwhile, C18:1n9, as oleic acid, claimed the highest value. It is then followed by polyunsaturated fatty acids (PUFA) (33.87%), where the major fatty acid in this group is linoleic acid (C18:2n6). Another compound of this group is C18:3n3, which although is a smaller portion, in terms of importance in human health, it is of great interest as an essential fatty acid. Eventually, saturated fatty acids showed the smallest share, with palmitic acid (C16:0) being the dominant compound.

In assessing the lipid class of membrane phospholipids, it was observed that the content of polar and neutral lipids of rapeseed lecithin was 51.75% and 49.24%, respectively. Phosphatidyl choline was the major constituent of phospholipids of rapeseed lecithin (32.89% ± 1.1), followed by phosphatidyl ethanol amine (11.69% ± 0.73) and phosphatidyl inositol (6.80% ± 0.98). It should be noted that 48.35% ± 1.3 of phospholipids of rapeseed were undetermined fractions.

### 3.2. Particle Size Analysis

The mean hydrodynamic diameters of nanoliposomes as a function of AC concentration are presented in Table 2. 

The particle size is one of the key factors playing a significant role in the stability of nanoparticles, extent of sedimentation, accumulation as well as biological functions including absorption, diffusion, biodistribution, and pharmacokinetics. Reduction of the particle size results in increased surface area to volume ratio and stability of colloidal systems [21]. As presented, the hydrodynamic diameter of the initial nanoliposomes was 140.74 nm, and with an elevation in the AC concentration, eventually, the size of particles increased to 194.67 nm. The results suggest that the encapsulated compound, here barberry anthocyanin extract, has a direct effect on the particle size of the colloidal suspension which is also observed in other studies [22,23,24]. Generally, the structure of bioactive compounds and their arrangement in the lipid bilayer membrane have a remarkable impact on particle size. As a consequence of the homogenization process, the layered structure of nanoliposome is altered into the vesicle ones. Therefore, anthocyanin molecules are distributed in the created molecular cavities of nanoliposomes. In fact, AC may entrap in the internal core and absorb onto the surface of nanoliposomes owning to hydrogen bonding (through hydroxyl groups of both mentioned species) as well as electrostatic interaction between the phosphate head groups of phospholipids and flavylium cation of AC. Additionally, AC can distribute in the double layers of the membrane as a result of hydrophobic interactions. Accordingly, the outcome of these interactions causes the size enlargement of particles. It is clear that the possibility of the mentioned interactions will rise with increasing the concentration of AC [24,25].

PDI is another important factor that indicates the formation quality of nanoliposomes whether mono (stable) or polydisperse. This dimensionless index considers the width of the diagram of particle size distribution. PDI less than 0.3 represents the monodisperse colloidal system. If this factor is less than 0.3, it is desirable, and the range of the diagram of particle size distribution is small and the stability of the system is acceptable [26]. In this study, PDI values were less than 0.3 for all samples, and in this regard, there was no significant difference between the samples. Nevertheless, we observed gradual growth of PDI with concentration. It is likely that, with an elevation in concentration, the content of AC that lies between membrane layers through hydrophobic interactions increases, eventually causing increased heterogeneity and PDI. 

### 3.3. Zeta Potential

The results of the zeta potential of unloaded and loaded nanoliposome samples are presented in Table 2. The surface charge of liposomes represents the amount of repulsion among charged nanoparticles and plays a prominent role in stability and encapsulation efficiency. It is initially contingent upon the charge of lipids, followed by the layers absorbed onto the surface, nature and composition of the environment in which liposomes are dispersed [23]. It is well-established fact that if the zeta potential value of samples is greater than +30 mV or less than –30 mV, due to the increased repulsive interactions, either electrostatic or steric, the system is stable and not prone to aggregation [23]. As can be observed in Table 2, the zeta potential of all samples was less than –42.85, suggesting high stability of the systems. The negative charge of nanoliposomes in this study was associated with anionic phospholipids including phosphatidyl choline and phosphatidyl ethanol amine within the structure of rapeseed lecithin (Section 3.1). In the current study, incorporation of AC extract to nanoliposomes results in a slight shift (<−10 mV) to a less anionic potential, but nevertheless, all samples have a similar zeta potential value indicating the formation of anionic, stable liposomes. The results obtained from the present study were in line with the research by Zhao et al., however, Guldiken et al. did not observe any changes in the zeta potential with the elevation of the concentration of AC [22,24]. Interaction between flavylium cation (present in anthocyanin structure) with phospholipids causes relative coating of the negative charge of nanoliposomes and their increased zeta potential. It is obvious that with the rise of the extract concentration, the magnitude of zeta potential and repulsive forces among particles would be diminished.

### 3.4. Encapsulation Efficiency (EE)

The results of the effect of concentration of AC on the EE are reported in Table 2. As can be observed, with the elevation in the AC concentration, EE increased, where the maximum level was observed for the concentration of 9% (47.19%). These results, enhanced EE with elevated concentration of bioactive compounds, have been reported in numerous studies [12,13,24]. EE is contingent upon numerous factors including the hydrophilic and lipophilic nature of bioactive compounds and the intensity of their interaction with the wall constituent compounds (here phospholipids). Further, the volume of the utilized water, membrane fluidity, and surface area of nanoparticles also affect EE [8,16,27]. As mentioned earlier, although AC are hydrophilic molecules to which a number of hydroxyl groups have been attached, these compounds also have relative hydrophobic characteristics because of possessing aromatic rings. Due to this special structure, characteristics such as EE and the size of nanoliposome particles containing these compounds are affected, causing their characteristics to be different from the properties of other hydrophilic compounds. Patently, the amphiphilic characteristics of AC due to their special structure leads to their arrangement in the aqueous nucleus and double layer membrane. As reported in Table 2, the variation of EE by AC concentration is well consistent with the nanoliposomes particle size. Since EE is in line with the volume of water entrapped within the structure of nanoliposomes, larger nanoliposomes cause an increased volume of the aqueous nucleus, culminating in enhanced EE of hydrophilic bioactive compounds. Therefore, with the elevation of extract concentration, the probability of electrostatic interaction between the flavylium cation and negatively charged phospholipids species in the structure of rapeseed lecithin increases as well as the relative positioning of AC in the nanoliposome bilayer, and thus, the reaction between the hydrophobic part of AC and fatty acyl chain of phospholipids increases, where the outcome of these interactions eventually leads to increased encapsulation efficiency [28].

As mentioned, the maximum EE was observed for nanoliposomes including 9% extract. On the other hand, these samples had the minimal absolute value of zeta potential, suggesting diminished surface charge and electrostatic repulsion between vesicles. Since these samples did not have suitable stability during storage time (data are not shown), in spite of their higher EE in comparison to the others, samples containing 4.5% were chosen for subsequent studies.

### 3.5. Stability of Nanoliposomes

The stability of unloaded and AC-loaded nanoliposome samples (containing 4.5% *w*/*v* AC) was evaluated relying on the variation of particle size, PDI, zeta potential, and EE. The results indicate no significant difference in the studied factors for unloaded nanoliposomes following 30 days of storage at both 4 and 37 °C (*p* > 0.05). A similar case was observed for AC-loaded nanoliposome at 4 °C, where no significant difference was observed in the measured parameters after 30 days (*p* > 0.05). However, the AC-loaded samples showed a different behavior at 37 °C. Although no significant difference was observed in the parameters of zeta potential and PDI at the end of storage time (*p* > 0.05), on the 20th day, the particle size and EE significantly increased and decreased respectively. This was in such a way that at the end of the storage period, particle size and EE were 188.9 nm and 32.67%, respectively. 

Predominantly, nanoliposomes are thermodynamically unstable colloidal systems in which the particle size and amount of releasing encapsulated compounds gradually increase. The instability of nanoliposomes can be attributed to chemical interactions, lipid oxidation, and aldehyde production as well as physical collisions of nanoparticles and membrane integration [12,29]. The physical collisions resulting from random motions of particles are controlled by the interactions between the particles of the colloidal system. As previously mentioned, in all of the samples, zeta potential did not show any significant changes during the storage period. Thus, the repulsive force between vesicles caused suspension stability [30]. Elevation of the size of particles in AC-loaded nanoliposomes is just due to physical collisions among nanoparticles. Since particle size of unloaded nanoliposomes did not change considerably in both studied temperatures, there is no possibility of particle development resulting from oxidation and production of aldehyde compounds. In fact, enlargement of particle size (AC-loaded nanoliposomes) at 37 °C is a consequence of the intrinsic low absolute value of zeta potential in these systems and thus diminished intensity of repulsive force among charged particles. High temperature leads to increased molecular mobility and coalescence among particles. Moreover, hydrophilic compounds usually lie in the internal aqueous phase of nanoliposomes, and thus they are subject to hydrolytic degradation [31]. This may lead to diminished encapsulation efficiency and indeed elevation the release of AC entrapped inside nanoliposomes over time.

### 3.6. Nanoliposome Morphology

**Figure 2:**
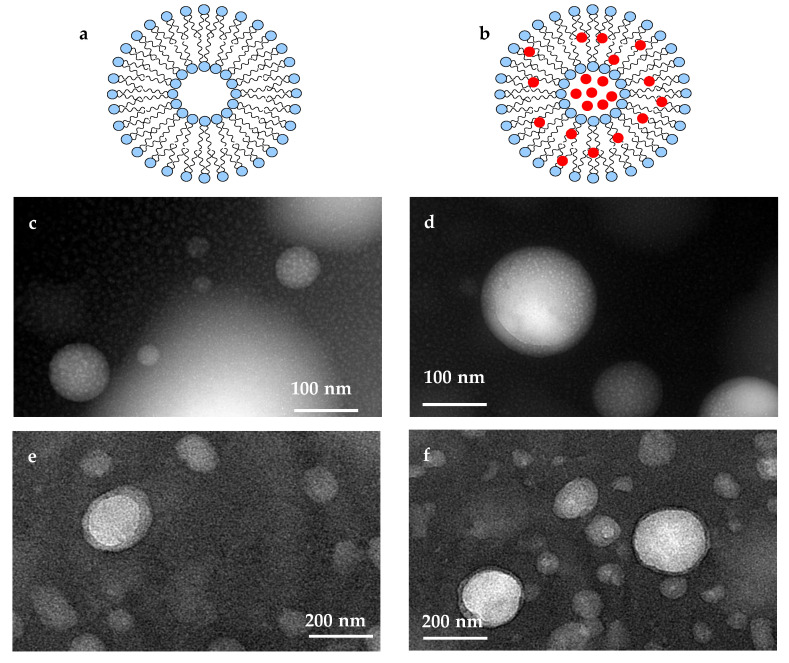
Schematic illustration of nanoliposomes: (**a**)—unloaded nanoliposomes, (**b**)—AC-loaded nanoliposomes; TEM images of nanoliposomes: (**c**)—unloaded nanoliposomes, (**d**)—AC-loaded nanoliposomes; TEM images of nanoliposomes representing the bilayer nature of them: (**e**)—unloaded nanoliposomes, (**f**)—AC-loaded nanoliposomes.

Morphologies of unloaded and AC-loaded nanoliposomes are presented in Figure 2. The results of TEM can provide visual information about the morphology of nanoliposomes particles. TEM images in Figure 2c,d indicate that the unloaded nanoliposome containing AC had a spherical and semi-spherical appearance. Trace amounts of oil drops in the colloidal suspension of nanoliposomes can be well observed in TEM images (the light points in Figure 2c). This is because employed rapeseed lecithin was not pure phospholipid. As illustrated, in comparison to LP-FR samples (Figure 2e), loading of AC resulted in an improved spherical state of nanoliposomes and thus their regulatory and symmetry (Figure 2f). These findings suggest that AC had a favorable effect on the packing of phospholipid bilayers [24]. The vesicles produced by the ultrasound method appeared layered, which is because of the sonication stage. This confirms that the nanoparticles are liposomes. Furthermore, the bilayer structure of nanoliposomes is well revealed in Figure 2e,f. 

### 3.7. Membrane Fluidity

In this study, the extent of the fluidity of the phospholipid membrane was 4.23 ± 0.07 and 3.73 ± 0.04 for unloaded and AC-loaded nanoliposomes respectively. Membrane fluidity refers to the relative extent of free mobility of the phospholipid chain of alkyls in liposome bilayers. Basically, the profile of releasing compounds from liposome structures is a function of two factors: the intensity of the interaction between the drug–liposome and bimolecular membrane fluidity. To a great extent, leakage of the drug to the surrounding aqueous solution increases with the elevation of the fluidity of this layer [9,32]. Therefore, it can be inferred that the loading of AC to the nanoliposome structure caused diminished membrane fluidity. This event is possibly due to the type of structure of AC and their interactions with phospholipids. In fact, because of the interaction with the head part of phospholipids and distribution in the hydrophobic part of the membrane, AC may have caused diminished fluidity of the lipid membrane. Hereupon, the presence of AC influences the packing characteristics of the phospholipid bilayers, considering the decreased membrane fluidity [16].

### 3.8. In Vitro Analysis of Biocompatibility of Liposomes

#### 3.8.1. Cellular Cytotoxicity Evaluation

**Figure 3:**
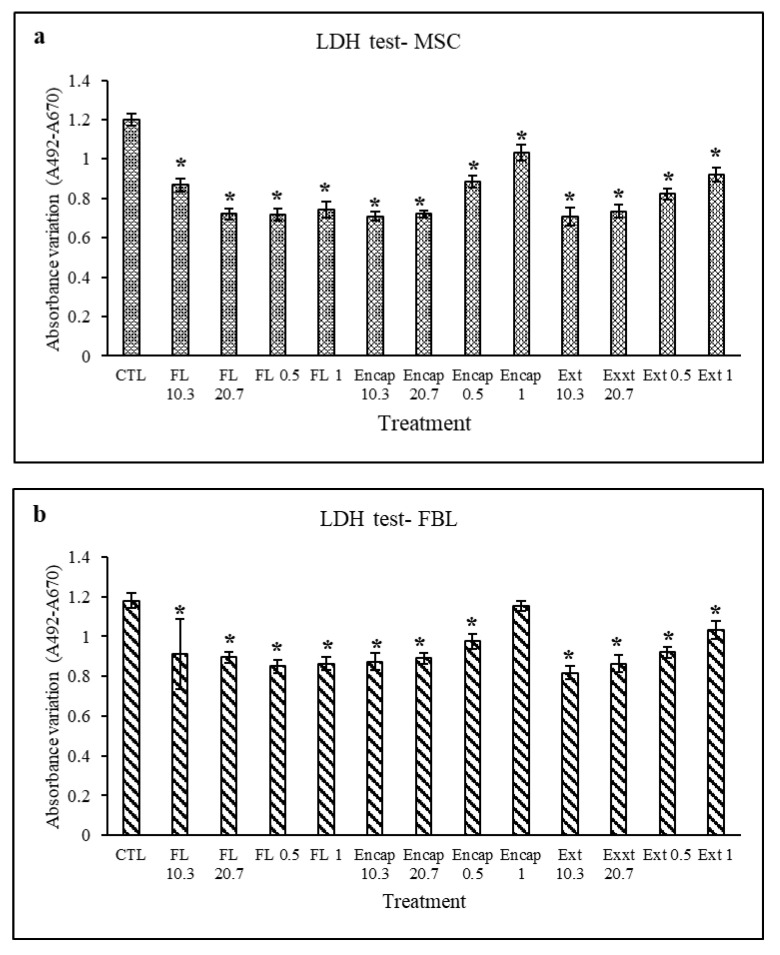
Lactate dehydrogenase activity of human cells: (**a**)—mesenchymal (MSC) cells, (**b**)—FBL cells under different treatments by: AC extract (Ext), unloaded nanoliposomes (FL) and AC-loaded nanoliposomes (Encap). CTL is control sample including unstimulated cells. Indexes of 1, 0.5, 20.7 and 10.3 represent the concentration of different treatments as 1 mg.mL^−1^, 0.5 mg.mL^−1^, 20.7 µg.mL^−1^, and 10.4 µg.mL^−1^ respectively. * indicates there is significant statistical difference between different treatments and control sample (*p* < 0.05). the results are mean of three replications ± SD.

The activity of extracellular lactate dehydrogenase enzyme was conducted for evaluating the cytotoxicity after stimulation of the human cells with different samples and various concentrations. Further, the control sample (CTL), containing unstimulated human cells, representing the basal level of LDH released by the untreated cells, was also considered in this test. As can be observed in Figure 3, in both types of tested cells, the LDH levels released from the control sample were lower or did not have any significant difference with the treated ones. These results suggest that AC extract and unloaded and loaded nanoliposomes lacked any cytotoxicity for both types of cells.

#### 3.8.2. The Metabolic Activity of FBL and MSC Cells

The MTT test allows for estimating the mitochondrial metabolic activity of cells. This test is based on the respiration of cells through which the activity of mitochondria dehydrogenase is measured. A high metabolic activity represents the proper functioning of cells [33]. 

**Figure 4:**
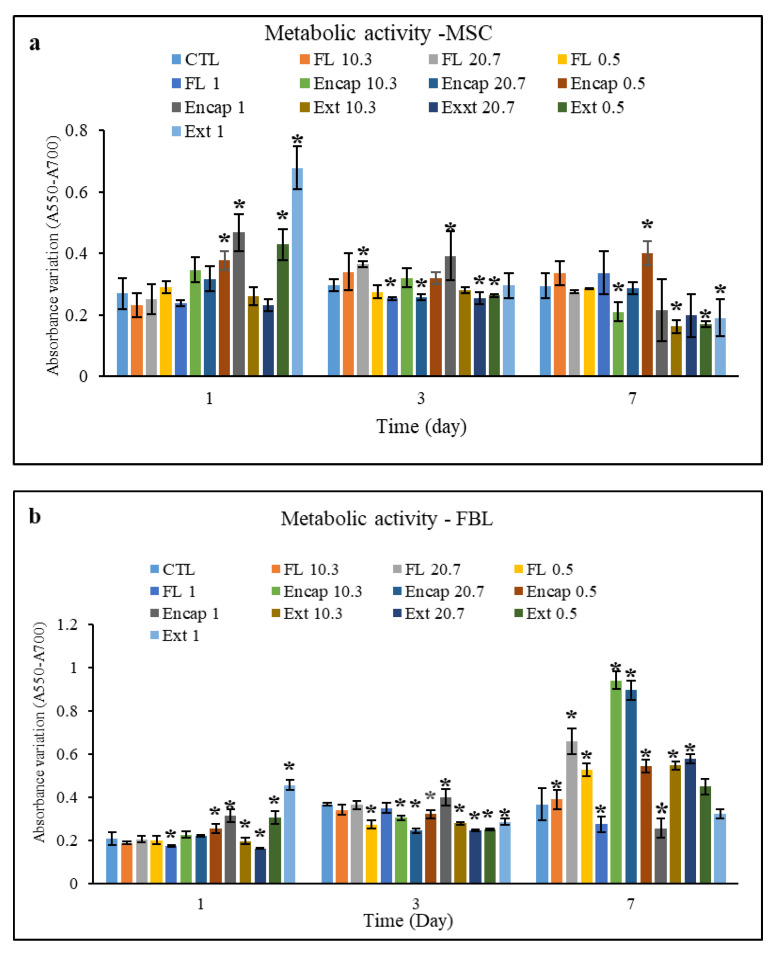
Metabolic activity of human cells: (**a**)—MSC cells, (**b**)—FBL cells under different treatments by: AC extract (Ext), unloaded nanoliposomes (FL) and AC-loaded nanoliposomes (Encap). CTL is control sample including unstimulated cells. Indexes of 1, 0.5, 20.7 and 10.3 represent the concentration of different treatments as 1 mg.mL^−1^, 0.5 mg.mL^−1^, 20.7 µg.mL^−1^, and 10.4 µg.mL^−1^ respectively. * indicates there is significant statistical difference between different treatments and control sample (*p* < 0.05). The results are mean of three replications ± SD.

The effect of different treatments on the metabolic activity of MSC cells is illustrated in Figure 4. As observed (Figure 4a), the metabolic activity, irrespective of the time of treatment, diminished over time, and only the samples Encap-0.5 and FL-1 showed an initial descending and then ascending trend. Additionally, the results of different treatments on metabolic activity FBL cells (Figure 4b) represent the general trend of metabolic activity of cells over time, which was ascending, and only Encap-1 samples first showed an ascending and then descending trend, while the Ext-1 sample revealed a diminishing trend. As stated above and shown in Figure 4, the maximum metabolic activity of FBL cells was observed in the Encap-10.3 treatment, while for MSC, it was observed in the Encap-0.5 treatment.

According to the acquired results, as mentioned earlier, the various employed treatments in this research had different effects in terms of being ascending, descending, and neutral concerning the cellular metabolic activity. On the whole, the MTT level is dependent on the cellular replication rate and indeed in the exponential growth phase, the reduction of the MTT level is greater than the stationary phase [34]. The descending trend of metabolic activity over time could be due to confluence in response to cell replication and phenotypical changes of cells. The last probability should be investigated by genomic tests and protein analysis [18]. Another hypothesis is the effects of the post-stimulation of cells. In this regard, they try to adapt themselves through long-term extracellular stimulation, thereby reducing metabolic activity [20].

Elevation of metabolic activity of the cells stimulated by unloaded nanoliposomes is due to the presence of PUFA (ω-3 and ω-6) and vitamin E, which has antioxidant properties, in rapeseed lecithin causing improved body functioning against the free radicals and enhanced metabolic activity of cells [20].

The higher metabolic activity of cells treated with AC extract is likely a result of its potent antioxidant effects. AC cause prevention from the development of reactive oxygen species inside mitochondria through reducing free radicals and deactivating them. Further, the greater metabolic activity of cells treated with AC-loaded nanoliposomes is a consequence of the synergistic effects of rapeseed lecithin and AC on metabolic activity [35].

#### 3.8.3. Cellular Proliferation of FBL and MSC Cells

**Figure 5:**
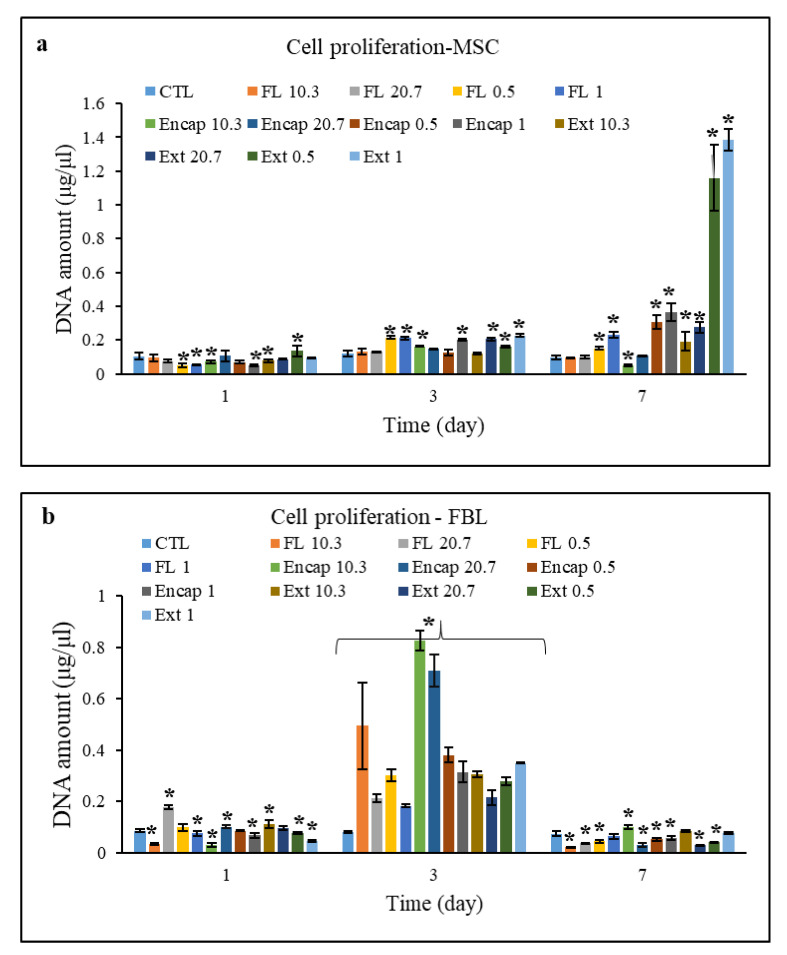
Cell proliferation of human cells: (**a**)—MSC cells, (**b**)—FBL cells under different treatments by: AC extract (Ext), unloaded nanoliposomes (FL) and AC-loaded nanoliposomes (Encap). CTL is control sample including unstimulated cells. Indexes of 1, 0.5, 20.7 and 10.3 represent the concentration of different treatments as 1 mg.mL^−1^, 0.5 mg.mL^−1^, 20.7 µg.mL^−1^, and 10.4 µg.mL^−1^ respectively. * indicates there is significant statistical difference between different treatments and control sample (*p* < 0.05). The results are mean of three replications ± SD.

The results of the effect of different treatments on the proliferation of FBL and MSC cells are demonstrated in Figure 5. As observed in Figure 5a, in all of the samples containing MSC cells, the DNA content increased over time, except for FL-10.3, FL-20.7, FL-0.5, Encap-10.3, and Encap-20.7, which had a descending trend on the seventh day.

Concerning the samples containing FBL (Figure 5b), an absolutely different trend was observed. All of the samples had a growing trend of DNA content compared to the first day, while on the seventh day, DNA content revealed a considerable diminishing trend. As illustrated, for FBL and MSC cells, samples Encap-10.3 and Ext-1 had the maximum DNA content and cellular proliferation respectively.

Elevation of cellular proliferation in the presence of AC is first due to their high antioxidant power and significantly improving the mitochondrial function for producing high amounts of bioenergy, as well as the presence of sugar compounds in the AC structure and promotion of metabolism of sugar compounds, causing better growth of cells [18]. On the other hand, these compounds protect the DNA against radicals thanks to their antioxidant properties [36]. Elevation of DNA content in the presence of unloaded nanoliposomes and certainly rapeseed lecithin is possibly due to the energy-generating role and ATP production throughout the cellular metabolism cycle. 

Note that the behavior of MSC and FBL cells varies in different treatments. This is possibly because different cells have different receptors for various bio-compounds, and thus they will influence the function of endocytosis of cells and diffusion of compounds [20].

As a consequence, the interaction of cells and biomolecules is a complex dynamic process, which depends on the type of cell and the properties of biomolecules. The results of experiments showed that metabolic activity, DNA content and proliferation of cells depend on the concentration of lecithin and biomolecule. Therefore, considering MTT tests and DNA content, it can be interpreted that the best formulation with the maximum biocompatibility and bioavailability for FBL and MSC cells was AC-loaded nanoliposome with concentrations of 10.3 µg/mL and 0.5 mg/mL, respectively. Note that except for MSC cells, Ext 1 treatment showed the maximum cellular proliferation, since these compounds are usually unstable in the gastrointestinal tract, in spite of their favorable bioactivity, they were not considered as the optimal formulation.

## 4. Conclusions

Liposomes are compatible phospholipid vesicles widely used in pharmaceuticals, food, and nutraceutical industries. Encapsulation of bioactive compounds causes their isolation and protection against external conditions, thereby improving chemical stability, bioavailability, and their health-promoting effects. In this study, the possibility of using liposomes composed of rapeseed lecithin for AC encapsulation was investigated, and the chemical characteristics of nanoliposomes were examined before and after encapsulation of the target compounds. The combination of hydration and ultrasound processes resulted in the formation of nanoliposomes with homogeneous particle size distribution. With an elevation in AC concentration, the size of particles and surface charge of the nanoparticles increased. Further, it was observed that there is a positive correlation between the size of nanoliposome particles and the encapsulation efficiency. Additionally, TEM image confirmed the sphericity of the produced particles, whose size lied within the nanoscale. In addition, diminished fluidity of phospholipid membranes of nanoliposomes in the presence of AC represented reduced release and linkage rate of bioactive compounds to their surroundings. The biocompatibility in vitro tests of nanoliposome samples were performed on FBL and MSC cell classes. The results of LDH tests showed that encapsulation of AC inside nanoliposomes did not cause any toxic effect on the mentioned cells. On the other hand, the results of MTT tests and DNA replication suggested improved metabolite activity and replication of cells in the presence of AC-loaded nanoliposomes, which is due to their antioxidant and energy generation activity. Therefore, it can be concluded that encapsulating AC as a valuable nutraceutical compound in nanoliposome causes both improved stability, and provides the possibility of their purposeful delivery in the sights of interest by optimizing their formulation. 

## Figures and Tables

**Table 1:** Main fatty acid composition of rapeseed lecithin.

Fatty Acids	Values (% ± SD)
C16:0	7.31 ± 0.01
C18:0	1.43 ± 0.03
C20:0	0.31 ± 0.02
C22:0	0.19 ± 0.01
**∑SFA**	9.24
C16:1 n-9	0.31 ± 0.02
C18:1 n-9	55.63 ± 0.09
C20:1 n-11	0.70 ± 0.04
C22:1 n-9	0.25 ± 0.04
**∑MUFA**	56.89
C18:2 n-6	27.32 ± 0.04
C18:3 n-3	6.55 ± 0.01
**∑PUFA**	33.87

**Table 2:** Mean particle size, PDI, zeta potential and encapsulation efficiency of fabricated nanoliposomes.

Concentration of ACs (%)	Particle Size (nm)	PDI	Zeta Potential (mV)	EE (%)
0	140.74 ± 4.05 ^d^	0.229 ± 0.02 ^a^	–48.77 ± 1.56 ^a^	-
4.5%	166.21 ± 3.01 ^c^	0.224 ± 0.02 ^a^	–45.55 ± 0.93 ^b^	40.18 ± 1.37 ^c^
6.75%	173.10 ± 2.74 ^b^	0.234 ± 0.01 ^a^	–43.88 ± 0.95 ^b,c^	45.56 ± 0.71 ^b^
9%	194.67 ± 6.34 ^a^	0.269 ± 0.04 ^a^	–42.85 ± 0.77 ^c^	47.19 ± 1.10 ^a^

All data are represented as mean ± SD. AC: anthocyanin compounds, PDI: polydispersity index, EE: encapsulation efficacy. Different letters (^a^, ^b^, ^c^, ^d^) reveal the significant differences (*p* < 0.05) between response variables as a function of Concentration of ACs (%).
